# Florid Pulmonary Mycobacterium avium-intracellulare Infection in a Patient With Large Granular Lymphocytic (LGL) Leukemia on Chronic Cyclophosphamide

**DOI:** 10.7759/cureus.19754

**Published:** 2021-11-19

**Authors:** Arsenije Kojadinovic, Prabhjot S Mundi

**Affiliations:** 1 Internal Medicine, Mount Sinai Medical Center, New York, USA; 2 Internal Medicine/Hematology-Oncology, Columbia University College of Physicians and Surgeons, New York, USA

**Keywords:** chronic t-cell leukemias, mycobacterium-avium intracellulare, pulmonary mac, opportunistic infection, large granular lymphocytic leukemia, lgl leukemia

## Abstract

Large granular lymphocytic (LGL) leukemia is a rare form of incurable chronic leukemia frequently complicated by life-threatening cytopenias. The less common NK-cell variant of this disorder poses a diagnostic challenge and its etiologic basis is poorly understood. Here we present the case of an elderly man diagnosed with LGL leukemia after presenting with severe Coombs-negative hemolytic anemia, who had a robust durable response to oral cyclophosphamide. Close to two years after initial diagnosis, he developed a florid *Mycobacterium avium-intracellulare* (MAI) infection of the lungs. We discuss the clinical and pathologic features of this case, highlighting aspects common to this disorder and areas of clinical uncertainty. We hope to both raise awareness of the risk for pulmonary MAI infection in patients treated with lymphodepleting drugs and to motivate the prospective evaluation of strategies to prevent opportunistic infections in LGL leukemia.

## Introduction

Large granular lymphocytic (LGL) leukemia is a rare form of incurable chronic leukemia with an annual incidence of 0.2 cases per million and a clinical spectrum ranging from asymptomatic disease to life-threatening cytopenias [1-2]. The median age at diagnosis is 66 years and about 50% of cases are associated with antecedent autoimmune disorders. The malignant clone is either a CD3+ effector T-cell or, less commonly, a CD3-/CD16+ natural killer (NK) cell.

The development of severe immune-mediated cytopenias heralds the need to initiate treatment. The antifolate, methotrexate, is the preferred first-line agent, but cyclophosphamide and cyclosporine A are also commonly used [3-5]. Here we present the case of an elderly male diagnosed with LGL leukemia after presenting with acute onset transfusion-dependent anemia, who had a robust response to oral cyclophosphamide but subsequently developed Mycobacterium avium-intracellulare (MAI) infection of the lungs.

## Case presentation

An 87-year-old white male with a history of extensive tobacco use, ST-elevation myocardial infarction, coronary artery bypass surgery, and chronic obstructive pulmonary disease (COPD) presented to the emergency department with two weeks of rapidly progressive lightheadedness and severe exertional dyspnea. A routine laboratory workup was most notable for a hemoglobin level of 6.7 gm/dL with an elevated mean corpuscular volume (MCV), representing a significant drop compared to 10 weeks prior to presentation when the hemoglobin level was 13.1 gm/dL with an MCV of 92.7 fl. The patient was admitted to the hospital for further workup and management.

Additional laboratory studies are summarized in Table 1. Pertinent findings included a decreased neutrophil count, elevated iron indices, including ferritin level, and evidence of hemolysis, including a suppressed haptoglobin level. The direct antiglobulin test was non-reactive for both immunoglobulin G (IgG) and complement.

**Table 1:** Initial laboratory studies at presentation

	Value	Normal Range	Units
Complete Blood Count			
White Blood Cell Count	4.3	4.8-10.8	thousands of cells per microliter (K/cmm)
Hemoglobin	6.7	14-18	grams/deciliter (gm/dL)
Hematocrit	19	42-52	percent
Mean Corpuscular Volume	99	80-96	femtoliters
Platelet Count	143	130-400	K/cmm
Lymphocyte Count	3	0.9-5.2	K/cmm
Neutrophil Count	0.8	1.9-6.0	K/cmm
Iron Panel			
Iron	210	42-135	micrograms/deciliter (mcg/dL)
TIBC	276	280-400	mcg/dL
Iron Saturation	76.1	15-50	percent
Ferritin	737.6	32-295	nanograms/milliliter (ng/mL)
Hemolysis Panel			
Haptoglobin	3	16-200	milligrams/deciliter (mg/dL)
Lactate Dehydrogenase	314	94-260	Units/Liter (U/L)
Reticulocyte Count	0.75	0.5-1.5	percent
Bilirubin	1.0	0.2-1.2	mg/dL
Misc			
B12	1557	210-920	picograms/milliliter (pg/mL)
Folate	12.8	5-18	ng/mL
Erythropoietin	87.1	2.6-18.5	milliunits per milliliter

The patient received three units of packed red blood cells (PRBCs) with a suboptimal response and underwent an urgent endoscopic evaluation that did not reveal an active source of bleeding. Hematology was consulted. A review of the peripheral blood smear demonstrated the presence of increased reticulocytes and a majority of lymphocytes with atypical features, including abundant vacuolated cytoplasm and prominent azurophilic granules (LGL cells). A bone marrow evaluation was pursued. Flow cytometry on the aspirate specimen confirmed the presence of an aberrant population of NK or atypical T-cells representing 26% of all nucleated cells, with an immunophenotype positive for CD2, CD7, CD11c, CD16, and CD56 and negative for surface and cytoplasmic CD3, TCRab, TCRgd, CD4, and CD8. CD57 was equivocal, positive on a small subset of these cells. On core biopsy, there was modestly increased cellularity with evidence of trilineage hematopoiesis without overt dysplasia, and the presence of patchy atypical CD2+/CD56+ lymphoid aggregates representing 15% of the total cellular component. There was no evidence of TCR-beta or gamma clonal rearrangement by polymerase chain reaction assays.

**Figure 1:**
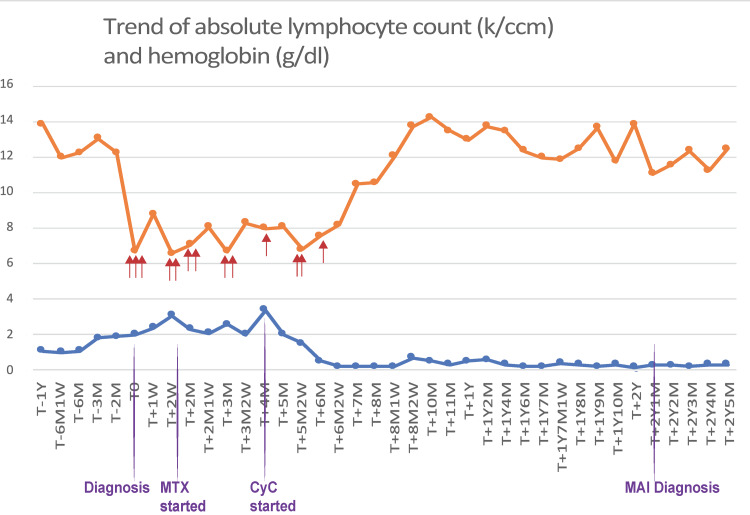
Hemoglobin and absolute lymphocyte count trend Hemoglobin (orange) and absolute lymphocyte count (blue) trend, in grams/deciliter and thousands of cells per microliter, respectively. T0 is time of presentation. The red arrows signify PRBC units transfused. The patient has been independent of transfusions starting at three months after cyclophosphamide initiation. Pulmonary MAI infection was diagnosed more than two years after the initial diagnosis. [D – days; M – months; Y – years; CyC – cyclophosphamide; MTX – methotrexate; PRBC – packed red blood cells; MAI – Mycobacterium avium-intracellulare]

The clinicopathologic findings were consistent with chronic NK-cell LGL leukemia. Oral methotrexate 7.5 mg/m^2^ weekly was started and subsequently increased to 12.5 mg/m^2^ weekly, along with darbepoetin 100 mcg biweekly given a suboptimally elevated erythropoietin level, and deferasirox for iron chelation. Unfortunately, the patient remained transfusion-dependent over the next three months, requiring approximately 2 units of PRBCs every two weeks in the clinic to maintain the hemoglobin level above 7 gm/dL (Figure 1). He also reported persistent exertional dyspnea in spite of the use of continuous supplementary oxygen. A transthoracic echocardiogram was notable for an estimated pulmonary artery systolic pressure of 80 mmHg, suggestive of severe pulmonary hypertension.

The decision was made to discontinue methotrexate and start cyclophosphamide 50 mg daily; prednisone 10 mg daily was also added. There was a notable decrease in transfusion dependency, and the dose of cyclophosphamide was further increased to 100 mg five days and 50 mg on two days each week. Approximately three months after the initiation of cyclophosphamide, the patient was transfusion independent, his exertional dyspnea had substantially improved, and he was rarely needing supplemental oxygen. The prednisone and darbepoetin were discontinued as the hemoglobin level increased to 14 gm/dL. The absolute neutrophil count had returned well into the normal range while the total lymphocyte count was consistently below 300 per uL, consistent with a complete hematologic response and non-specific lymphodepletion. He received twice-weekly trimethoprim-sulfamethoxazole for *Pneumocystis jirovecii *prevention [6]. The patient opted to continue this regimen beyond 12 months after discussing the potential long-term risks of cyclophosphamide use, which include myeloid neoplasm and bladder cancer [7].

**Figure 2:**
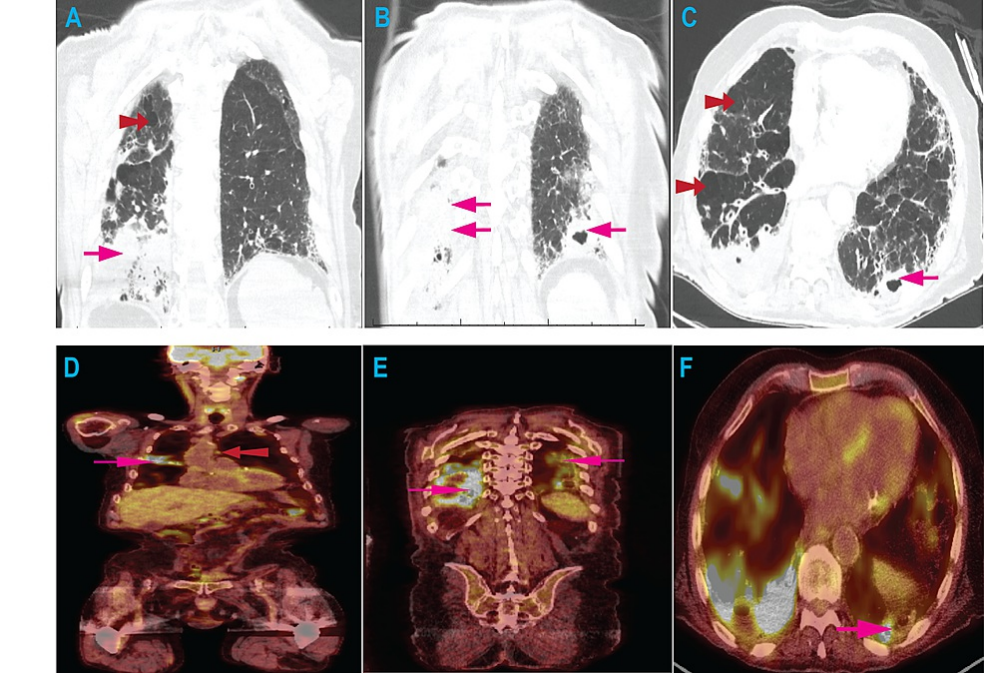
Radiologic findings A and B. CT scan chest (coronal view). Dense infiltrates (pink arrows) are present in a predominant posterior distribution, most pronounced in the right lower lobe. There are also several large bullae (red double arrowheads), consistent with centrilobular and paraseptal emphysematous changes. The infiltrates were not present on a CT scan two years earlier. C. CT scan chest (axial view). A 3.0 cm thick-walled cavitary lesion is seen in the left lower lobe base (pink arrow), also seen in panel B. D and E. 18-FDG PET scan (coronal view, anterior and posterior sections). There are intense streaks and patches of 18-FDG uptake (pink arrows) with maximum standardized uptake values (SUV) ranging from 29 to 45, significantly brighter than the physiologic uptake in the myocardium. Other regions within the infiltrate have minimal metabolic activity. The bilateral hilar and mediastinal lymph nodes have modestly increased uptake, SUV <5 (red double arrowhead). F. PET scan chest (axial view). There is significant metabolic activity in part of the rim of the cavitary lesion (pink arrow). FDG – fluorodeoxyglucose; PET – positron emission tomography

Two years after initial presentation, the patient reported two months of progressive cough with copious whitish sputum along with anorexia and weight loss. The complete blood count continued to indicate remission of the LGL leukemia and ongoing suppressed lymphocyte count. A computed tomography scan demonstrated bilateral dense mass-like consolidations most pronounced in the posterior lower lobes and bilateral hilar and mediastinal lymphadenopathy measuring up to 1.5 cm (Figures 2A-2C). A cavitary component was noted in the left lower lobe. Given the concern for possible malignancy, 18-fluorodeoxyglucose (FDG) positron emission tomography (PET) was performed and demonstrated patchy foci of severe hypermetabolic activity (maximum standardized uptake values of 29 to 45) in the lung parenchyma and modest activity in the lymph nodes (Figures 2D-2F).

There was a clinical concern for opportunistic infection. Induced sputum specimens were collected and acid-fast bacillus smears demonstrated three (3) to four-plus (4+) mycobacterium. Polymerase chain reaction (PCR)-based testing confirmed non-tuberculous Mycobacterium avium-intracellulare. After discussing the potential adverse effects of anti-mycobacterial therapies, he started a regimen that includes daily azithromycin, ethambutol, and rifampin. The cyclophosphamide dose was decreased to 50 mg daily. Three months after initiating anti-mycobacterial therapy, the sputum cultures have cleared but there continue to be ongoing symptoms of anorexia and depressed mood. The LGL leukemia remains in remission.

## Discussion

LGL leukemia is a rare malignancy characterized by immune-mediated cytopenias, most commonly neutropenia (80%), anemia (50%), and splenomegaly (50%) [8]. It is postulated that chronic immune stimulation, directed to either a self-antigen or a yet-to-be-identified viral infection, may drive the initial clonal expansion of terminal effector T-cells. Subsequent malignant transformation requires escape from normal Fas-mediated activation-induced cell death through a DAP10/DAP12-dependent mechanism [9]. Indeed, the discovery of recurrent clonal TCR rearrangements across patient samples supports this hypothesis for T-cell LGL.

The NK-cell variant of LGL leukemia is less commonly associated with antecedent autoimmune disorders and its etiologic basis is poorly understood. While our patient had a 30-year history of osteoarthritis, he had negative titers for rheumatoid factor, anti-cyclic citrullinated peptide, and antinuclear antibodies. Definitively establishing the clonality of the NK cells is formidable, although the monotypic expression of killer Ig-like receptor isoforms (KIR; CD158a/b/c) may be helpful [10-12]. The presence of recurrent STAT3 and STAT5B mutations in 30% to 40% of both T- and NK-cell LGL implies shared pathobiology but in and of themselves are not diagnostic [13-14].

The median survival after diagnosis with LGL leukemia is close to 10 years, with most deaths not directly attributable to the disease. Nevertheless, there is considerable morbidity and early deaths can occur. In larger case series, 10%-20% of patients succumb within two years of diagnosis, the majority due to mucocutaneous and pulmonary infections with typical bacterial pathogens in the setting of refractory neutropenia [3,15]. About 80% of patients will require treatment at some point. Objective response rates on the order of 55%-70% have been demonstrated in small prospective studies of methotrexate, cyclophosphamide, and cyclosporine, although complete responses are less common with cyclosporine [1]. Smaller case series have also demonstrated good responses to the purine analogs pentostatin and fludarabine [16-17]. The optimal sequencing and duration of therapy have not been prospectively evaluated.

We believe that the lymphodepleting effects of cyclophosphamide, as well as the presence of severe obstructive lung disease, were the main contributing factors to our patient developing MAI infection [18]. Cyclophosphamide impacts immune function through both a myelosuppressive effect and direct inhibition of effector cell function. As a case in point, major bacterial and fungal infections are reported in the absence of neutropenia in 5%-21% of patients on prolonged oral cyclophosphamide treatment for granulomatosis polyangiitis or lupus nephritis [19-20]. Pulmonary hypertension, which is associated with LGL leukemia but also occurs frequently in individuals with COPD, is not known to increase the risk of infection 

Innate drug resistance is common in MAI, with treatment failure in up to 40% of cases not associated with HIV, resulting in high mortality rates [21]. The choice of discontinuing cyclophosphamide versus continuing treatment at a lower dose was weighed carefully. Given the limited available treatment options and the severe impact LGL leukemia previously had on his quality of life, we decided to continue cyclophosphamide.

## Conclusions

We hope this case both raises awareness of the risk for pulmonary MAI infection in patients treated with lymphodepleting drugs and motivates the prospective evaluation of optimal strategies to prevent opportunistic infections in LGL leukemia. Two potential preventative measures would be to switch to intermittent maintenance dosing when remission is achieved or to monitor CD4+ or total lymphocyte counts and initiate azithromycin prophylaxis when below specific thresholds.
